# Food Safety Culture and Education: A Cross-Sectional Study in Southern Italy

**DOI:** 10.3390/foods14234095

**Published:** 2025-11-28

**Authors:** Piersaverio Marzocca, Vito Cerabona, Vincenzo Marcotrigiano, Umberto Farina, Teresa Tarricone, Ylenia Tatoli, Michele Lampedecchia, Giacomo Domenico Stingi, Caterina Spinelli, Maria Grazia Forte, Roberto Rizzi, Annamaria Dalena, Sandro Cinquetti, Giovanni Normanno, Maria Teresa Montagna, Christian Napoli, Osvalda De Giglio, Giuseppina Caggiano

**Affiliations:** 1 Prevention Department, Food Hygiene and Nutrition Service, Local Health Authority Bari, 70056 Bari, Italy; piersaverio.marzocca@asl.bari.it (P.M.); umberto.farina@asl.bari.it (U.F.); ylenia.tatoli@asl.bari.it (Y.T.); caterina.spinelli@asl.bari.it (C.S.); mariagrazia.forte@asl.bari.it (M.G.F.); 2 Department of Medicine and Surgery, University of Perugia, 06129 Perugia, Italy; vito.cerabona@unipg.it; 3 Prevention Department, Local Health Authority “ULSS 1 Dolomiti”, 32100 Belluno, Italy; sandro.cinquetti@aulss1.veneto.it; 4 Prevention Department, Food Hygiene and Nutrition Service, Local Health Unit BT, 76125 Trani, Italy; teresa.tarricone@aslbat.it (T.T.); michele.lampedecchia@aslbat.it (M.L.); giacomodomenico.stingi@aslbat.it (G.D.S.); 5 Prevention Department, Local Health Authority Taranto, 74121 Taranto, Italy; roberto.rizzi@asl.taranto.it (R.R.); annamaria.dalena@asl.taranto.it (A.D.); 6 Department of Sciences of Agriculture, Food, Natural Resources and Engineering, University of Foggia, 71122 Foggia, Italy; giovanni.normanno@unifg.it; 7 Interdisciplinary Department of Medicine, Hygiene Section, University of Bari Aldo Moro, 70124 Bari, Italy; mariateresa.montagna@uniba.it (M.T.M.); osvalda.degiglio@uniba.it (O.D.G.); giuseppina.caggiano@uniba.it (G.C.); 8 Department of Medical Surgical Sciences and Translational Medicine, Sapienza University of Rome, 00189 Rome, Italy; christian.napoli@uniroma1.it

**Keywords:** food safety culture, public health, prevention department, food business operators, training, healthcare workers, official controls

## Abstract

At present, food safety culture (FSC) is a strategic dimension of food management systems, as established by EU Regulation 2021/382. Understanding how individual and organizational factors influence the safety climate is essential for designing effective training interventions. A cross-sectional observational study was conducted in Southern Italy from March to September 2025 among 216 workers from 52 food businesses located in the provinces of Bari and Barletta-Andria-Trani, using a questionnaire based on a validated scale, which is structured into five dimensions of leadership, communication, commitment, risk awareness, and resources. Descriptive analyses, χ^2^ tests, and multivariate logistic regressions were conducted to identify predictors of positive perceptions. The overall climate was positive for 82.9% of the sample. The strongest dimensions were leadership (88.4%), communication (86.1%), and risk awareness (84.7%), while resources were identified as a critical area (41.2% of respondents perceived deficiencies, especially in personnel and infrastructure). Overall, the results confirm that FSC is significantly influenced by ongoing training, cultural awareness and job stability. Resource-related challenges indicate a need for greater management investment to concretely support food safety practices.

## 1. Introduction

Food safety is one of the main priorities of European health policies and a fundamental pillar of public health. With the entry into force of Regulation (EU) 2021/382, the community legislator introduced significant innovations in the regulatory framework related to food hygiene, focusing on risk management, food redistribution, and above all, the promotion of food safety culture (FSC) [1].

The latter, in line with the European Commission Communication (2022/C 355/01), is now recognized as an essential component of food safety management systems (FSMSs), aimed at promoting an informed and participatory approach by Food Business Operators (FBOs) [2]. Over the last two decades, harmonized European legislation has favored the adoption of management systems based on good hygiene practices, hazard analysis and critical control points (HACCP). Scientific evidence shows that these approaches have improved the quality and safety of food produced in Europe, but the increasing complexity of supply chains and the evolution towards a circular economy pose new challenges and emerging risks for food safety [3].

Recent studies, such as the one conducted by De Boeck et al., highlight how the analysis of FSC through structured models allows for the evaluation of the level of awareness of FBOs, correlating behavioral aspects with the overall performance of FSMSs [4]. In particular, a distinction is made between FSC, which is understood as the set of shared values, beliefs and behaviors that permanently guide an organization’s approach to food safety, and food safety climate, which represents its most contingent and perceived dimension at the moment, i.e., a “snapshot” of workers’ perceptions of leadership, communication, commitment, the availability of resources, and risk awareness. This distinction allows for the interpretation of culture as a reference framework that remains stable over time and climate as the dynamic and daily manifestation of this culture. The proposed approach integrates the evaluation of objective elements, linked to management systems and official controls, with the analysis of subjective perceptions of staff, allowing for the identification of any misalignments that may impact the overall effectiveness of FSMSs [5,6,7].

Food businesses require a deeper analysis of FSC, which has so far been unexplored in detail. For this reason, it becomes important to understand the organizational, managerial, and operational reasons behind the methods of FBOs, who could have forgotten all theoretical grasps over time. The meaning of culture includes all these facets. Specifically, the role of training, work experience, and knowledge of the FSC concept represent areas that need to be carefully explored, as the behaviors adopted by food personnel can have direct repercussions on the healthiness of food products, whether they are ingredients or ready-to-eat foods.

At the national level, the Puglia Region stands out for its strong agri-food vocation and the widespread presence of food businesses in the primary and secondary sectors. Regional legislation—in particular Regional Law no. 22/2007 and Regulation no. 5/2008—governs the training of FBOs, promoting the adoption of good hygiene practices and procedures compliant with the HACCP system. These provisions are consistently aligned with the Regional Prevention Plan (PRP) 2021–2025, which incorporates the objectives of the National Prevention Plan (PNP) 2020–2025, oriented towards the One Health approach [8]. This paradigm recognizes the interconnection between human, animal, and environmental health and identifies food safety culture as a key element in reducing the risk of foodborne diseases and promoting public health.

FSC refers to organizational culture theory, safety climate theory, and behavioral change theory, according to which safety depends not only on codified procedures but also on the organization’s ability to encourage preventive behaviors, shared responsibility, and continuous learning. Hence, an effective FSC requires consistent leadership commitment, transparent internal communication, continuous process monitoring, and regular staff training such that the prevention of any contaminant becomes a priority and is internalized at every level of the organization. Taking all these aspects into account, this study arises from the need to assess the degree of implementation of Regulation (EU) 2021/382 in the Apulian manufacturing sector and to analyze the level of cultural maturity of food businesses regarding food safety, helping to bridge the gap between the regulatory and behavioral dimensions of food safety. The central research hypothesis is that there is a significant correlation between the level of training of FBOs and the perception of FSC within food businesses. It is also hypothesized that food businesses with more attentive leadership, more effective internal communication, and greater risk awareness have a more developed FSC and manage health and hygiene risks more efficiently.

## 2. Materials and Methods

This cross-sectional observational study was conducted in Southern Italy in the northern macro-area of the province of Bari and the province of Barletta-Andria-Trani (BAT). The study population consisted of FBOs operating in 52 food businesses subjected to official controls between March and September 2025 by the Competent Local Health Authorities, specifically the Food Hygiene and Nutrition Services of the Bari Local Health Authority (Northern Area) and the BT Local Health Authority. Overall, the target population included 479 FBOs. Sample size calculation was therefore based on these data, with a 95% confidence level and a 5% margin of error, assuming *p* = 0.5 (maximum variability). Applying finite population correction, the minimum required sample size was determined to be 214 participants, ensuring both adequate statistical power and adequate representativeness.

The businesses included in the study operated in a variety of food sectors and represented the main business categories in the northern macro-area of the province of Bari and the province of Barletta-Andria-Trani (BAT). Based on their primary business, they were grouped into five macro-categories, as defined by the European Commission Communication (2022/C 355/01):

i. Production/Processing: 12 food businesses, 174 FBOs (36.3%);
ii. Public Catering: 20 food businesses, 92 FBOs (19.2%);
iii. Collective Catering: 6 food businesses, 116 FBOs (24.2%);
iv. Retail: 7 food businesses, 64 FBOs (13.4%);
v. Wholesale/Distribution: 6 food businesses, 47 FBOs (9.8%);
vi. Other: 1 food business, 1 FBO (0.2%).

Specifically, i. refers to registered or approved food establishments producing food intended for commercialization; ii. refers to bars, pizzerias, restaurants, and similar food establishments, including those within hotels and other accommodations; iii. concerns cooking centers and canteens in schools, hospitals, retirement homes, and similar settings; iv. refers to all retail businesses where consumers make direct purchases (e.g., minimarkets, supermarkets, and hypermarkets); v. includes sales not intended for consumers but to other FBOs, who in turn market the food; and vi. extends the food chain to other categories not included in the first five (e.g., food trucks, home-food and home-restaurants).

The sample design adopted is random: the food businesses surveyed were identified following the planning included in the annual risk-based control plans of both Local Health Authorities, based on the risk categorization, which takes into account many aspects, including type of food, size of the company, marketing area, time distance from the most recent inspection, and results of the last control. In terms of company size, most food businesses were classified as small and medium-sized enterprises based on the number of employees and operating space.

A structured questionnaire was developed, inspired by the contents of the European Commission Communication (2022/C 355/01). The instrument was organized into four sections: the first (6 items) explored sociodemographic characteristics; the second (8 items) addressed aspects related to professional experience; the third (8 items) focused on training; and the fourth (28 items) assessed FSC. The fourth section was further subdivided into five domains: leadership, communication, commitment, risk awareness and resources. These items reflect the fundamental components through which an organization can assess how its employees perceive the internal climate regarding hygiene and food safety.

The questionnaire was administered as part of official inspection activities conducted by both Local Health Authorities: during inspections, healthcare workers (HCWs) invited FBOs to participate in the study by completing an anonymous questionnaire, accessible via a QR code. Participation was voluntary and anonymous. Respondents could complete the questionnaire on site, immediately after the inspection, or remotely within a specified period using a unique access link. Informed consent was provided electronically prior to participation. No personal identifiers were collected, ensuring confidentiality and data protection in accordance with the General Data Protection Regulation (EU) 2016/679.

Data were collected through a web-based survey administered via the Microsoft Forms^®^ platform.

The collected data were analyzed using descriptive statistical methods for each section and subsection of the questionnaire. Differences between groups were assessed using the chi-square test, applied to categorical variables. Stratified analyses were performed to explore variations between homogeneous subgroups and to control for potential confounding factors. Associations between sociodemographic and occupational variables and the dimensions of De Boeck et al.’s scale were assessed using logistic regression models. The scale was adopted as it is, without any change. Estimates were expressed as ORs with corresponding 95% confidence intervals (95% CIs). The significance level was set at 0.05. Covariates were selected for inclusion in the multivariate model based on a *p*-value < 0.25 in the univariate analysis or their significance. Several sociodemographic variables were coded and categorized to facilitate statistical analysis. Gender was classified into four categories: female, male, non-binary, and “preferred not to answer”. Age was initially recorded as five cohort groups (<25, 25–34, 35–44, 45–54, ≥55 years) and subsequently aggregated into three macro-categories: young (<35 years), adult (35–54 years), and senior (≥55 years). Marital status was divided into four categories (married, single, cohabiting, widowed) and subsequently dichotomized into married vs. never married. Education level was classified into three levels: basic (no education or lower secondary school qualification), intermediate (high school diploma), and university (bachelor’s degree or equivalent, including a PhD). Italian language proficiency was divided into three categories: basic (scale 1–3), intermediate (4–7), and native speaker (8–10). Seniority was initially recorded as five classes (<1 year, 1–5, 5–10, 10–20, >20 years) and then aggregated into two categories (<10 vs. ≥10 years).

The type of contract (permanent, fixed-term, apprenticeship, entrepreneur, freelancer) was grouped into employees and self-employed/entrepreneurs. Work commitment was classified as part-time or full-time. Food businesses’ characteristics were also coded: the type of activity (originally nine categories) was grouped into four macro-categories (production, distribution/catering, healthcare/consultancy, and other). Company size was assessed based on both the number of employees and the surface area, classified as small, medium, or large. Job role was classified into six categories (production, quality, office, sales, technical, entrepreneur/other) and subsequently aggregated into three macro-categories: operational, technical/professional, and managerial. The geographical area was coded as Bari (northern area) or BAT. Training-related variables included training frequency (regular, occasional, unknown, none), delivery method (face-to-face, remote, blended), perceived adequacy (adequate, somewhat adequate, not entirely adequate, unknown, not provided), participation in allergen risk training courses (yes/no), knowledge of food safety culture (yes/no), and the perceived impact of one’s work on food safety (low = 1–3, medium = 4–7, high = 8–10). Food safety culture was assessed according to the scale by De Boeck et al., which covers five key dimensions: leadership, communication, commitment, risk awareness and resources. For each dimension, responses were rated on a 5-point Likert scale (1 = strongly disagree, 5 = strongly agree). Dimensional scores were obtained by summing the relevant items, while categorical levels (low, medium, high) were defined based on predefined cut-offs. Finally, each dimension was dichotomized, grouping low and medium levels (coded as 0) and high levels as the reference category (coded as 1). Analyses were performed using STATA 18 statistical software (StataCorp LLC, TX, USA).

This study was conducted in accordance with the Declaration of Helsinki and approved by the Ethics Committee of the University of Bari Aldo Moro (session of Research Ethics Committee, protocol No. 0076191, approval date 6 March 2025).

## 3. Results

### 3.1. Sociodemographic Characteristics of Sample

The questionnaire was distributed to 52 food businesses in two provinces of the Puglia Region, 32 in the northern macro-area of the province of Bari, and 20 in the province of Barletta-Andria-Trani. Of the 479 eligible participants invited to participate in the survey, 216 FBOs completed the questionnaire, achieving a response rate of 45.1%. The main sociodemographic and occupational characteristics of the study population are summarized in Table 1. The sample consisted of 216 respondents, of whom 55.1% were male, 44% female, and 0.9% non-binary. The predominant age group was 35 to 54 (44%), followed by younger participants under 35 (41.2%) and older participants over 55 (14.8%). Marital status was almost evenly distributed, with 51.8% single and 48.2% married. Regarding educational level, almost half of the participants reported an intermediate level of education (48.6%), while 31.5% had a university degree and 19.9% had only basic education (middle school). Knowledge of the Italian language was generally high: 75.5% classified themselves as native speakers, 17.6% as intermediate, and 6.9% as basic. Seniority was well balanced, with 54.2% reporting less than 10 years of service and 45.8% reporting more than 10 years. Most participants were employed (91.2%) rather than self-employed (8.8%), with a prevalence of full-time contracts (69%). Most of the companies represented operated in the distribution/catering sector (75.5%) and were predominantly small (44.4% with fewer than 10 employees and up to 250 m^2^ of space). Regarding company roles, operational staff constituted the largest group (52.8%), followed by management (35.1%) and technical–professional roles (12.1%). Geographically, 60.2% of respondents stated that they worked in the northern macro-area of the province of Bari, while 39.8% carried out their business in the province of Barletta-Andria-Trani. Regarding in-company food safety training, 70.4% stated that they received it periodically, while 57.4% deemed it adequate and 12.5% deemed it not entirely adequate. Training was provided primarily in person (68.5%) and most often on an annual basis (53.7%). Furthermore, more than half of the respondents (54.6%) attended specific courses on allergen risk management. Finally, 75% said that they had heard of the concept of FSC. Perceptions of the impact of their work on food safety were generally high: 65.3% considered it very relevant, 32.4% moderate, and only 2.3% believed that their contribution is of little relevance.

### 3.2. De Boeck et al.’s Levels of Food Safety Dimensions and General Climate

**Figure 1:**
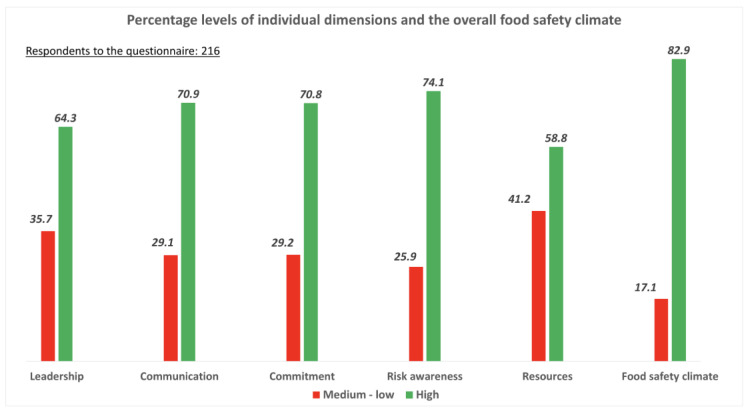
Percentage levels of individual dimensions and De Boeck’s overall food safety climate.

The analysis of the scores obtained using the De Boeck et al. scale (Figure 1) provides an overall positive picture of the food safety climate. Regarding leadership, 64.3% of participants reported high levels, while 35.7% placed it in the medium–low range. Similar distributions were observed for communication and commitment, with 70.9% and 70.8% of respondents, respectively, reporting high levels, compared to approximately 29% reporting medium–low levels. Risk awareness emerged as the most consolidated dimension: 74.1% of participants indicated a high level, while only 25.9% fell into the medium–low category. Conversely, the perception of resources represented the most critical area. Although 58.8% of participants rated this dimension positively, more than four in ten (41.2%) perceived the available resources as insufficient or suboptimal. Considering the overall index, the general food safety climate was rated as high by 82.9% of participants, with 17.1% giving it medium–low ratings.

### 3.3. Univariate Analysis

Chi-square analysis revealed several significant associations with the leadership and communication dimensions (Table 2). For leadership, age was significantly correlated (*p* = 0.035): older individuals (>55 years) were more likely to report high scores (78.1%) compared to younger participants (<35 years; 54.1%). Marital status also showed a significant association (*p* = 0.022), with higher levels among married respondents (72.1%) compared to unmarried respondents (57.1%). A particularly strong relationship was observed with length of service (*p* < 0.001): employees with more than 10 years of experience reported high leadership scores 77.8% of the time, compared to 53% of those with less experience. Education emerged as a key contributing factor: regular participation in food safety training was strongly associated with higher leadership scores (77%), while the percentage dropped to 37.5% among those who received occasional training and 12.5% among those who did not receive training (*p* < 0.001). Similarly, the perceived adequacy of the training was highly significant (*p* < 0.001): 7.8% of those who considered it adequate reported high scores, compared to 33.3% of those who judged it “not entirely adequate”. The training delivery method was also significant (*p* = 0.007): in-person training produced higher scores (68.9%) than distance training (52.6%), and scores were lower among those who could not answer the question (23.1%). The frequency of training activities confirmed this trend (*p* = 0.001), with annual training associated with high scores among 75% of respondents, compared to 37.5% giving high scores for three-year cycles and 43.6% for four-year cycles. Other significant associations were participation in specific courses on allergen risk management (72% vs. 55.1%; *p* = 0.010) and familiarity with the concept of FSC (72.8% vs. 38.9%; *p* < 0.001). Finally, the perception of the impact of one’s work on food safety was strongly associated (*p* = 0.001): 72.3% of those who rated their impact highest (8–10) reported high leadership scores, compared to 20% of those who rated their impact lowest (1–3). For communication, similar associations were observed. Furthermore, age was also significant (*p* = 0.042): the percentage of high scores progressively increased from 61.8% among younger respondents to 75.8% among adults and 81.2% among older adults. Marital status was also associated (*p* = 0.028), with higher scores among married participants (77.9% vs. 64.3% for unmarried).

As with leadership, seniority showed a strong correlation (*p* < 0.001): 85.9% of those with more than 10 years of experience reported high scores, compared to 58.1% of those with less experience. Training was again a key factor: regular food safety training was associated with significantly higher communication scores (82.2%) than occasional training (46.4%) or no training (25%) (*p* < 0.001). Similarly, the adequacy of training was strongly associated (*p* < 0.001): 81.4% of those who rated it as adequate reported high scores in the communication domain, compared to 63.8% of those who rated it as “somewhat adequate” and 44.4% of those who rated it as “not at all adequate”. The frequency of training events confirmed this trend (*p* = 0.001): annual training produced the highest percentage of high scores (82.8%), which decreased with less frequent training (59% biennial, 43.7% triennial, 61.5% quadrennial). Other associations included participation in allergen risk management courses (78% vs. 62.2%; *p* = 0.011) and prior knowledge of FSC (77.8% vs. 50%; *p* < 0.001). Finally, the perception of the impact of one’s work on food safety was highly significant (*p* < 0.001): only 20% of those who rated their impact lowest reported high communication scores, compared to 58.6% in the middle range and 78.7% in the highest range.

Significant associations emerged between commitment and risk awareness and several sociodemographic variables (Table 2). Married participants reported high levels of commitment (78.8%) and risk awareness (81.7%), significantly more often than unmarried participants (63.4% and 67%, respectively; both *p* = 0.013). Length of service also played an important role: employees with more than 10 years of experience showed higher levels of commitment (80.8%) and risk awareness (81.8%), compared to those with shorter service (62.4% and 67.5%; *p* = 0.003 and *p* = 0.017, respectively). Similarly, the contractual framework influenced the results: full-time workers were more likely to report high commitment (76.5%) and risk awareness (78.5%) than part-time workers (58.2% and 64.2%; *p* = 0.006 and *p* = 0.026). Another determining factor was the type of role held, with management reporting the highest levels of commitment (81.6%) and risk awareness (84.2%), compared to those in operational roles (64% and 66.7%; *p* = 0.033 and *p* = 0.024). Company size also showed an effect on commitment: workers in companies larger than 500 m^2^ recorded higher scores (81.4%, *p* = 0.033); for risk awareness, the trend was similar but did not reach significance (*p* = 0.089). The strongest associations were observed for training-related variables. Regular food safety training was strongly associated with greater commitment and risk awareness (82.9% and 84.9%), while occasional or no training produced much lower scores (both *p* < 0.001). Perceived adequacy of training further strengthened this pattern: commitment reached 84.7% among those who rated it adequate, but only 58.6% among those who rated it “fairly adequate” and 44.4% among those who rated it “not at all adequate” (*p* < 0.001). Risk awareness followed a similar trend (85.5%, 67.2%, and 40.7%; *p* < 0.001).

Delivery method also had a significant impact on engagement (*p* = 0.001), with higher scores reported for in-person training (77%) compared to distance learning (52.6%). For risk awareness, the difference did not reach statistical significance (*p* = 0.082). Training frequency showed a significant effect on engagement (*p* = 0.013): annual training was associated with the highest percentage of high scores (79.3%), followed by biennial (69.2%), triennial (43.7%), and quadrennial (61.5%) cycles. For risk awareness, the trend was similar but not statistically significant (*p* = 0.098). Participation in allergen risk management courses was significantly associated with commitment (79.7% vs. 60.2%; *p* = 0.002), while the association for risk awareness was not significant (*p* = 0.158). Knowledge of the concept of FSC was strongly related to both dimensions (*p* < 0.001): high levels of commitment (80.2%) and risk awareness (80.9%) were significantly more frequent among participants familiar with the concept than among those who were not (42.6% and 53.7%). Finally, self-perception of one’s actions as impacting food safety emerged as a significant predictor. For the commitment dimension, none of the participants in the lowest self-rating range (1–3) reported high scores, compared to 51.4% in the middle range (4–7) and 83% in the highest range (8–10; *p* < 0.001). Risk awareness followed a similar gradient (20%, 62.9%, and 81.6%; *p* < 0.001).

The analysis of resources and food safety climate shown in Table 2 demonstrates that, among the sociodemographic factors, age played a decisive role (*p* = 0.012): only 47.2% of younger participants (<35 years) reported high scores, compared to 65.3% of adults (35–54 years) and 71.9% of older participants (>55 years). Marital status also influenced the results (*p* = 0.006), with higher values among married respondents (68.3%) compared to unmarried respondents (50%). Length of service remained highly relevant (*p* = 0.001): participants with more than 10 years of work experience achieved higher scores in 70.7% of cases, while those with less experience (<10 years) achieved higher scores in only 48.7% of cases. Significant associations also emerged by company size: both the number of employees (*p* = 0.044) and surface area (*p* = 0.049) were associated with higher scores, which was more evident in large companies (>50 employees and >500 m^2^). Role type further contributed to the differences (*p* = 0.011), with individuals in managerial positions achieving higher values (72.4%) than respondents with operational roles (51.7%). Training-related variables confirmed their central role: receiving specific food safety training was strongly associated with higher scores (*p* < 0.001): 73% of regularly trained people achieved high levels, compared to only 25% of those trained occasionally or not at all. Delivery method was also important (*p* = 0.004): in-person training was associated with higher levels (66.2%) than distance learning or blended learning. The frequency of training events further strengthened this gradient (*p* < 0.001): annual training corresponded to the highest values (72.4%), while three-year (18.8%) and four-year (43.6%) cycles were much lower. Furthermore, self-perception of the impact of one’s work on food safety showed a clear gradient (*p* < 0.001): high scores were reported by 68.1% of those in the highest range (8–10), but only by 42.9% in the medium range (4–7) and 20% in the lowest range (1–3). Regarding the general food safety climate, many participants (82.9%) scored high, while only 17.1% fell into the medium–low category. No significant differences were observed regarding gender, age, marital status, education, or knowledge of the Italian language. It is worth noting that seniority showed a strong effect (*p* = 0.004): 90.9% of respondents with more than 10 years of experience reported a positive climate, compared to 76% of those with less experience. Role type was equally significant (*p* = 0.005), with management reporting higher levels (93.4%) than operational staff (75.4%). Training-related variables confirmed their criticality: regular training was associated with a positive climate in 91.4% of cases, compared to 66.1% of occasional interns and only 37.5% of those without training (*p* < 0.001).

### 3.4. Logistic Regressions

Perceptions of leadership impacting the food safety climate are shaped by both training experiences and professional background (Table 3). A key determinant is knowledge of FSC: respondents who report awareness are almost three times more likely to perceive leadership positively (OR = 2.78; 95% CI: 1.28–6.04; *p* = 0.011). The adequacy of training also proves highly influential. Participants who rate the training as “fairly adequate” are significantly less likely to report strong leadership (OR = 0.38; 95% CI: 0.18–0.80; *p* = 0.011), and the likelihood decreases further when training is rated as “not quite adequate” (OR = 0.31; 95% CI: 0.10–0.97; *p* = 0.045). The frequency of training was also central: compared to regular provision, occasional training significantly reduced the likelihood of a high perception of leadership (OR = 0.43; 95% CI: 0.19–0.97; *p* = 0.043), while the absence of training exerted a negative effect (OR = 0.07; 95% CI: 0.01–0.77; *p* = 0.029). Finally, seniority emerges as a protective factor: employees with more than ten years of service report significantly higher odds of perceiving leadership as strong (OR = 2.68; 95% CI: 1.32–5.43; *p* = 0.006), underlining the role of professional experience in strengthening this dimension.

Effective communication within the food safety climate correlates mainly with cognitive awareness and experience. As shown in Table 3, familiarity with FSC is a crucial factor: those who report such knowledge are almost three times more likely to perceive high levels of communication than those who are not aware of it (OR = 2.95; 95% CI: 1.39–6.28; *p* = 0.005). The regularity of training proves to be equally decisive: occasional attendance significantly reduces the likelihood of high levels of communication (OR = 0.31; 95% CI: 0.15–0.63; *p* = 0.001), while absence further amplifies this negative effect (OR = 0.09; 95% CI: 0.01–0.53; *p* = 0.008). Seniority also plays an important role: employees with more than ten years of experience are significantly more likely to communicate strongly than colleagues with a shorter tenure (OR = 3.82; 95% CI: 1.80–8.07; *p* < 0.001).

Training, professional role, and awareness are associated with levels of commitment to the food safety climate (Table 3). The frequency of training stands out as a key predictor: compared to those who regularly attend refresher courses, occasional participants are significantly less likely to report high commitment (OR = 0.20; 95% CI: 0.09–0.41; *p* < 0.001), and this probability decreases further in the absence of training (OR = 0.06; 95% CI: 0.01–0.37; *p* = 0.002). Professional role is also very important: employees in managerial positions are three times more likely to show high commitment than operational staff (OR = 2.71; 95% CI: 1.23–5.97; *p* = 0.013). The quality of training also contributes the following: rating it as “fairly adequate” is associated with significantly lower odds of high commitment (OR = 0.37; 95% CI: 0.18–0.79; *p* = 0.010). Finally, awareness of FSC emerges as a powerful factor, increasing the likelihood of strong commitment almost fourfold (OR = 3.94; 95% CI: 1.88–8.26; *p* < 0.001).

Risk awareness is strongly shaped by training and professional role (Table 3). The frequency of training is particularly influential: compared to workers who regularly attend courses, those trained occasionally report significantly lower odds of high awareness (OR = 0.31; 95% CI: 0.13–0.69; *p* = 0.004), while the absence of training reduces this probability to minimal levels (OR = 0.08; 95% CI: 0.02–0.36; *p* = 0.001). The adequacy of training is also a determining factor: participants who rated it as “fairly adequate” were less likely to report high awareness (OR = 0.37; 95% CI: 0.17–0.81; *p* = 0.013), with an even stronger negative effect for those who rated it as “not entirely adequate” (OR = 0.25; 95% CI: 0.08–0.73; *p* = 0.011). The role played within the organization also proved significant: professionals holding management roles were more than twice as likely to show high risk awareness compared to operational staff (OR = 2.56; 95% CI: 1.16–5.62; *p* = 0.020).

The perception of resource adequacy in the food safety climate is strongly influenced by variables related to training, knowledge of FSC, and professional role is described in Table 3. Workers who only occasionally benefit from training are significantly less likely to perceive resources as adequate than those who receive regular updates (OR = 0.15; 95% CI: 0.07–0.33; *p* < 0.001). Similarly, employees who rate training as “fairly adequate” show a significantly lower probability of giving positive evaluations (OR = 0.44; 95% CI: 0.21–0.93; *p* = 0.032). Conversely, knowledge of FSC significantly increases the likelihood of perceiving resources as sufficient (OR = 4.49; 95% CI: 2.03–9.93; *p* < 0.001). Training frequency plays an additional role: participants trained on a three-year basis are significantly less likely to perceive resources as adequate than those trained annually (OR = 0.18; 95% CI: 0.04–0.77; *p* = 0.020).

The overall food safety climate shows a multifactorial pattern. Seniority is a strong positive predictor: employees with more than ten years of service are more than three times more likely to report a favorable climate than their less experienced colleagues (OR = 3.33; 95% CI: 1.26–8.79; *p* = 0.015). Managers are more than four times more likely to provide positive evaluations than operators (OR = 4.39; 95% CI: 1.33–14.51; *p* = 0.015). Those who attended refresher courses every three years reported a lower probability of perceiving a positive climate (OR = 0.22; 95% CI: 0.06–0.78; *p* = 0.019), while even more infrequent refresher courses, such as one-off or five-yearly courses, further reduced this probability (OR = 0.15; 95% CI: 0.02–0.98; *p* = 0.047). A complete lack of training had the strongest negative impact (OR = 0.06; 95% CI: 0.01–0.40; *p* = 0.003). Knowledge of FSC stood out as a decisive protective factor, more than quadrupling the probability of perceiving the overall climate positively (OR = 4.44; 95% CI: 1.79–11.00; *p* = 0.001) (Table 3).

## 4. Discussion

Our study results highlight how the food safety climate is generally perceived positively: dimensions such as leadership, communication, commitment, and risk awareness show high percentages of “high” responses, and the overall climate is also judged favorable by 82.9% of the sample. However, the perception of resources is a critical factor: over 41% of participants reported deficiencies or insufficiencies in this area. This element represents a structural factor that can limit the effectiveness of FSMS, especially in sectors characterized by seasonality or high variability of workloads.

Univariate analyses highlight how personal and work-related factors, including age, marital status, length of service, and contract type, significantly influence the perception of climate across different domains. Seniority is confirmed as one of the most robust predictors: those who have worked for more than 10 years tend to have more positive perceptions of both leadership and communication, as well as commitment, awareness, and resources. This suggests that consolidated work experience contributes to the development of awareness, trust, and understanding of operational practices related to food safety.

Training appears to be a crucial and transversal element: not only its frequency (regular vs. occasional or absent) but also the perception of its quality has a marked influence on all dimensions of the climate. In particular, the absence of training or its evaluation as “not entirely adequate” drastically reduces the probability of perceiving a good climate, in line with recent systematic reviews showing how training courses have an impact on knowledge, attitudes, and safety practices [9]. The literature confirms that training must be continuous, relevant, and contextual to positively influence the FSC [6].

Another significant result concerns knowledge of the FSC: participants who declare familiarity with this concept have a much higher probability of perceiving a positive climate in almost all dimensions. This is consistent with what is reported in recent research, where leadership, organizational commitment, and shared culture emerge as central determinants in the promotion of safe environments [10,11]. These results are superimposable to those emerged in recent studies conducted in our country [12,13].

The logistic regressions applied in our study reinforce this evidence: managerial roles and seniority emerge as stable positive predictors, while a lack of training or a negative perception of it are strong penalizing factors. Knowledge of the FSC is confirmed as a protective factor, significantly increasing the probability of positive evaluations. These results are part of a consolidated line of studies that recognize that consistent managerial practices, effective communication, and continuous training are essential for consolidating an organizational food safety culture [14]. Further recent studies reinforce the relevance of these factors: for example, a critical analysis of the contribution of food culture to foodborne illnesses identified training and managerial support as essential levers for preventing risky behaviors, and that the implementation of FSMSs in food businesses from diverse contexts led to an improvement in leadership and the culture itself in a considerable number of cases [15,16,17].

A critical aspect concerns the area of resources: in small and medium-sized production facilities, lack of personnel, not adequate equipment or variable operating margins are recurring issues; furthermore, infrastructure investments are often limited. The observations collected suggest that in the food businesses, the sustainability of initiatives targeted at FSC strengthening does not depend exclusively on the quality of training programs or the presence of effective leadership but requires a constant commitment to maintaining the conditions necessary for the functioning of the FSMS and its subsequent sustenance. It can therefore be deduced that, in the absence of these conditions, even a favorable internal climate may be insufficient and may result in the inconsistent application of procedures, constituting an obstacle to the full maturation of the cultural climate [18].

Another point worthy of attention concerns the variability of perceptions expressed by younger workers. Indeed, their responses appear less stable than those of their more experienced colleagues, especially for areas related to communication and risk perception. This difference could lead not only to less exposure to complex operational situations, but also to the need for better-structured induction and mentoring programs, which allow for the progressive internalization of prevention practices. Arrangements not always designed for long-term contracts could also negatively impact, as they lead to a continuous turnover of the workforce, being unfavorable for workers from internalizing the basic concepts necessary for fine-tuning their skills and knowledge of operational processes. The homogeneity of assessments observed in senior groups seems to confirm the stabilizing role of experience.

The practical implications of our findings are as follows:

i. Training programs must take into account the company’s actual needs and the critical issues of the entire management structure, with a view to continuity over time. They cannot, therefore, be considered isolated events. It is also essential that they be delivered through interactive methods that encourage feedback and adaptation to operational needs.
ii. The active and visible involvement of management figures is essential if they are to serve as role models, enabling them to strengthen the credibility of food safety culture and stimulate employee interest.
iii. Companies must concretely evaluate and strengthen human, material, and time resources so that operational personnel are able to effectively apply rules and procedures.
iv. It is useful to adopt continuous climate assessment and monitoring tools, using various methodologies (surveys, observations, audits) that also allow for timely intervention and rapid modification of training methods should they prove ineffective.

Concretely, FSC is operationalized through the systematic translation of hygienic–sanitary principles into shared organizational practices and everyday behaviors, by encouraging staff empowerment and setting a good example, starting with managers. First, corporate leadership must adopt an exemplary stance and actively promote the culture by embedding it within strategic planning and decision-making processes. It requires continuous, predominantly practice-oriented training, aimed at ensuring widespread awareness of risks and control procedures. Appropriate internal communication sustains operational awareness, while the definition and measurement of standardized behaviors foster compliance with procedures. Equally central is the responsible engagement of all FBOs, supported by mechanisms that encourage the reporting of critical issues without fear of repercussions. FSC is grounded in a continuous cycle of review and improvement, based on internal audits, performance monitoring, and management of non-compliances. Moreover, the simplification of procedures and their alignment with the operational setting are essential to ensure the effective implementation of requirements and the long-term sustainability of food safety practices.

Our findings confirm that FSC is not built solely through technical procedures but requires an integrated ecosystem in which consistent training, active leadership, adequate resources, and a shared culture support each other. This integrated approach is consistent with the latest international evidence and represents an operational guide for companies intending to transform FSC from a mere formality to a strategic pillar of their operations, also considering new frontiers and global public health challenges which assess the potential correlation, including through HCWs, between workers and environmental characteristics associated with food safety culture [19,20,21].

Furthermore, our study highlighted that sustainability of food safety practices depends on organizations’ ability to maintain a consistent balance over time between shared culture, available resources, operational responsibilities, and a sense of belonging that can only arise from the genuine involvement of all workforces, regardless of the type of contract. Therefore, this balance becomes essential for translating cultural orientations into reliable and stable habits, as well as adopting behaviors based on a proactive rather than reactive approach.

In light of the findings from our study, future perspectives for improving and strengthening the FSC should consider the following: i. strengthening leadership and workers involvement, extending throughout the entire supply chain; ii. improving internal communication and information sharing; iii. digitalization and technological innovation, useful for predicting risks and automating controls at critical points; iv. a proactive, data-driven approach. Furthermore, the development of FSC will require a strengthening of collaborative initiatives across the entire agri-food supply chain, involving shared standards and inter-organizational monitoring mechanisms, involving both Local Health Authorities and Food Safety Agencies. Increasing attention is expected to be devoted to consumers, through initiatives aimed at enhancing risk awareness, strengthening trust, and promoting the active participation of citizens in food safety processes.

Although the results obtained provide significant insights into the food safety climate, this study has some limitations that deserve consideration. First, the cross-sectional design does not allow for the establishment of causal relationships but only associations between variables. Furthermore, the use of self-completed questionnaires carries a risk of potential social desirability bias and self-assessment errors, which may result in an overestimation of positive perceptions. Although the choice to recode the variables on a binary scale was appropriate for statistical analysis, it reduced the variability of the responses, introducing a risk of failing to capture more subtle information on nuances of the food safety climate. Finally, the specificity of the sample, which was limited to a specific geographical context, limits the generalizability of the results. As a result, caution is required when extending the conclusions to different organizational settings. It follows that further studies will need to be conducted to extend the findings to a national context.

## 5. Conclusions

Our findings indicate that FSC is largely viewed in a positive light. Increasing FSC is a prerequisite for maintaining food safety throughout the entire food supply chain. The benefits of adequate, timely training and defined retraining, combined with the management leadership style adopted in food businesses and the experience of FBOs, are key factors that significantly impact both the working environment, understood as worker well-being, and the culture of food safety. Prospectively, improving FSC will require a greater focus on workers, through more participatory leadership and ongoing training, supported by clear, two-way internal communication. The main goal is to build a more collaborative, transparent, and prevention-oriented system, involving all the actors in the complex of supply chains.

## Figures and Tables

**Table 1:** Sociodemographic characteristics of sample.

Variables	N	%
**Gender (216)**		
Female	95	44
Male	119	55.1
Non-binary	2	0.9
**Age (Classes)**		
Young people (<35 years)	89	41.2
Adults (35–54)	95	44
Senior (>55)	32	14.8
**Marital status**		
Unmarried	112	51.8
Married	104	48.2
**Education level**		
Basic	43	19.9
Intermediate	105	48.6
University	68	31.5
**On a scale from 1 to 10 (where 1 represents “basic level” and 10 “Madrelingua”), how do you rate your knowledge of the Italian language?**		
Basic (1–3)	15	6.9
Intermediate (4–7)	38	17.6
Madrelingua (8–10)	163	75.5
**Length of service**		
Less than 10 years	117	54.2
More than 10 years	99	45.8
**Type of employment contract**		
Dependent	197	91.2
Self-employed/Entrepreneur	19	8.8
**Contractual framework**		
Part-time	67	31
Full-time	149	69
**Company type**		
Production	42	19.4
Distribution/Catering	163	75.5
Consultancy	4	1.9
Other	7	3.2
**Company size (No. of employees)**		
Small (1 to 10 employees)	96	44.4
Medium (11 to 50 employees)	59	27.3
Large (>50 employees)	61	28.3
**Company size (sqm)**		
Small (up to 250 sqm)	96	44.4
Medium (from 250 to 500 sqm)	50	23.2
Large (>500 sqm)	70	32.4
**Type of role played in the food business**		
Operative	114	52.8
Technical–Professional	26	12.1
Management	76	35.1
**Territory**		
Northern macro-area of the province of Bari	130	60.2
Province of Barletta Andria Trani	86	39.8
**Do you receive specific training on food safety?**		
Yes, regularly	152	70.4
Yes, occasionally	56	25.9
No	8	3.7
**Do you think it is adequate?**		
Yes, it is adequate	124	57.4
Quite adequate	58	26.9
Not entirely adequate	27	12.5
I don’t know how to answer	4	1.8
Not existing	3	1.4
**Training Methods**		
In person	148	68.5
At a distance	19	8.8
Both modes	36	16.7
I don’t know how to answer	13	6
**How often do you receive training?**		
Annual	116	53.7
Biennial	39	18.1
Triennial	16	7.4
Every four years	39	18.1
Other	6	2.7
**Have you ever participated in specific training courses on allergen risk management (e.g., gluten)?**		
No	98	45.4
Yes	118	54.6
**Have you ever heard of FSC?**		
No	54	25
Yes	162	75
**On a scale of 1 (a little) to 10 (a lot), how much do you think your work can affect food safety?**		
Low (1–3)	5	2.3
Medium (4–7)	70	32.4
Very much (8–10)	141	65.3

**Table 2:** Associations between leadership, communication, commitment, risk awareness, resource size and overall food safety climate scale and sociodemographic/educational characteristics of sample.

	Leadership			Communication			Commitment			Risk Awareness			Resources			Food Safety Climate		
	Medium-Low	High		Medium-Low	High		Medium-Low	High		Medium-Low	High		Medium-Low	High		Medium-Low	High	
**Gender (216)**	N (%)	N (%)	*p*-value	N (%)	N (%)	*p*-value	N (%)	N (%)	*p*-value	N (%)	N (%)	*p*-value	N (%)	N (%)	*p*-value	N (%)	N (%)	*p*-value
Female	31 (32.6)	64 (67.4)	0.130	26 (27.4)	69 (72.6)	0.727	27 (28.4)	68 (71.6)	0.086	19 (20)	76 (80)	0.174	35 (36.8)	60 (63.2)	0.142	13 (13.7)	82 (86.3)	0.371
Male	44 (36.9)	75 (63.1)		36 (30.2)	83 (69.8)		34 (28.6)	85 (71.4)		36 (30.3)	83 (69.7)		52 (43.7)	67 (56.3)		24 (20.2)	95 (79.8)	
Non-binary	2 (100)	0		1 (50)	1 (50)		2 (100)	0		1 (50)	1 (50)		2 (100)	0		0	2 (100)	
**Age (Classes)**																		
Young people (<35 anni)	40 (44.9)	49 (54.1)	***0.035***	34 (38.2)	55 (61.8)	***0.042***	33 (37.1)	56 (62.9)	0.052	30 (33.7)	59 (66.3)	0.090	47 (52.8)	42 (47.2)	***0.012***	18 (20.2)	71 (79.8)	0.548
Adults (35–54)	30 (31.6)	65 (68.4)		23 (24.2)	72 (75.8)		25 (26.3)	70 (73.7)		19 (20)	76 (80)		33 (34.7)	62 (65.3)		15 (15.8)	80 (84.2)	
Senior (>55)	7 (21.9)	25 (78.1)		6 (18.8)	26 (81.2)		5 (15.6)	27 (84.4)		7 (21.9)	25 (78.1)		9 (28.1)	23 (71.9)		4 (12.5)	28 (87.5)	
**Marital status**																		
Unmarried	48 (42.9)	64 (57.1)	***0.022***	40 (35.7)	72 (64.3)	***0.028***	41 (36.6)	71 (63.4)	***0.013***	37 (33)	75 (67)	***0.013***	56 (50)	56 (50)	***0.006***	20 (17.9)	92 (82.1)	0.768
Married	29 (27.9)	75 (72.1)		23 (22.1)	81 (77.9)		22 (21.2)	82 (78.8)		19 (18.3)	85 (81.7)		33 (31.7)	71 (68.3)		17 (16.4)	87 (83.6)	
**Education level**																		
Basic	18 (41.9)	25 (58.1)	0.420	13 (70.2)	30 69.8)	0.713	12 (27.9)	31 (72.1)	0.929	12 (27.9)	31 (72.1)	0.917	16 (37.2)	27 (62.8)	0.769	8 (18.6)	35 (81.4)	0.947
Intermediate	33 (31.4)	72 (68.6)		28 (26.7)	77 (73.3)		30 (28.6)	75 (71.4)		26 (24.8)	79 (75.2)		43 (41)	62 (59)		18 (17.1)	87 (82.9)	
University	26 (38.2)	42 (61.8)		22 (32.3)	46 (67.7)		21 (30.9)	47 (69.1)		18 (26.5)	50 (73.5)		30 (44.1)	38 (55.9)		11 (16.2)	57 (83.8)	
**Italian language level**																		
Base	4 (26.7)	11 (73.3)	0.432	5 (33.3)	10 (66.7)	0.468	5 (33.3)	10 (66.7)	0.675	4 (26.7)	11 (73.3)	0.995	5 (33.3)	10 (66.7)	0.467	3 (20)	12 (80)	0.919
Intermediate	11 (28.9)	27 (71.1)		8 (21.1)	30 (78.9)		13 (34.2)	25 (65.8)		10 (26.3)	28 (73.7)		13 (34.2)	25 (65.8)		7 (18.4)	31 (81.6)	
Madrelingua	62 (38.1)	101 (61.9)		50 (30.7)	113 (69.3)		45 (27.6)	118 (72.4)		42 (25.8)	121 (74.2)		71 (43.6)	92 (56.4)		27 (16.6)	136 (83.4)	
**Length of service**																		
Less than 10 years old	55 (47)	62 (53)	***<0.001***	49 (41.9)	68 (58.1)	***<0.001***	44 (37.6)	73 (62.4)	***0.003***	38 (32.5)	79 (67.5)	***0.017***	60 (51.3)	57 (48.7)	***0.001***	28 (24)	89 (76)	***0.004***
More than 10 years old	22 (22.2)	77 (77.8)		14 (14.1)	85 (85.9)		19 (19.2)	80 (80.8)		18 (18.2)	81 (81.8)		29 (29.3)	70 (70.7)		9 (9.1)	90 (90.9)	
**Type of contract**																		
Dependent	73 (37.1)	124 (62.9)	0.164	60 (30.5)	137 (69.5)	0.179	61 (31)	136 (69)	0.061	53 (26.9)	144 (73.1)	0.291	83 (42.1)	114 (57.9)	0.372	36 (18.3)	161 (81.7)	0.151
Self-employed/Entrepreneur	4 (21.1)	15 (78.9)		3 (15.8)	16 (84.2)		2 (10.5)	17 (89.5)		3 (15.8)	16 (84.2)		6 (31.6)	13 (68.4)		1 (5.3)	18 (94.7)	
**Contractual framework**																		
Part-time	28 (41.8)	39 (58.2)	0.206	22 (32.8)	45 (67.2)	0.426	28 (41.8)	39 (58.2)	***0.006***	24 (35.8)	43 (64.2)	***0.026***	31 (46.3)	36 (53.7)	0.311	13 (19.4)	54 (80.6)	0.552
Full-time	49 (32.9)	100 (67.1)		41 (27.5)	108 (72.5)		35 (23.5)	114 (76.5)		32 (21.5)	117 (78.5)		58 (39)	91 (61)		24 (16.1)	125 (83.9)	
**Company type**																		
Production	16 (38.1)	26 (61.9)	0.632	12 (28.6)	30 (71.4)	0.658	10 (23.8)	32 (76.2)	0.850	9 (21.4)	33 (78.6)	0.904	21 (50)	21 (50)	0.535	8 (19.1)	34 (80.9)	0.630
Distribution/Catering	59 (36.2)	104 (63.8)		48 (29.4)	115 (70.6)		50 (30.7)	113 (69.3)		44 (27)	119 (73)		64 (39.3)	99 (60.7)		28 (17.2)	135 (82.8)	
Consultancy	1 (25)	3 (75)		2 (50)	2 (50)		1 (25)	3 (75)		1 (25)	3 (75)		2 (50)	2 (50)		1 (25)	3 (75)	
Other	1 (14.3)	6 (85.7)		1 (14.3)	6 (85.7)		2 (28.6)	5 (71.4)		2 (28.6)	5 (71.4)		2 (28.6)	5 (71.4)		0	7 (100)	
**Company size (No. of employees)**																		
Small (1 to 10 employees)	39 (40.6)	57 (59.4)	***0.050***	31 (32.3)	65 (67.7)	0.071	30 (31.2)	66 (68.8)	0.264	28 (29.2)	68 (70.8)	0.058	45 (46.9)	51 (53.1)	***0.044***	19 (19.8)	77 (80.2)	0.086
Medium (11 to 50 employees)	24 (40.7)	35 (59.3)		21 (35.6)	38 (64.4)		20 (33.9)	39 (66.1)		19 (32.2)	40 (67.8)		27 (45.8)	32 (54.2)		13 (22)	46 (78)	
Large (>50 employees)	14 (22.9)	47 (77.1)		11 (18)	50 (82)		13 (21.3)	48 (78.7)		9 (14.8)	52 (85.2)		27 (27.9)	44 (72.1)		5 (8.2)	56 (91.8)	
**Company size (sqm)**																		
Small (up to 250 sqm)	40 (41.7)	56 (58.3)	0.053	31 (32.3)	65 (67.7)	0.109	30 (31.2)	66 (68.8)	***0.033***	31 (32.3)	65 (67.7)	0.089	48 (50)	48 (50)	***0.049***	20 (20.8)	76 (79.2)	0.392
Medium (from 250 to 500 sqm)	20 (40)	30 (60)		18 (36)	32 (64)		20 (40)	30 (60)		13 (26)	37 (74)		19 (38)	31 (62)		8 (16)	42 (84)	
Large (>500 sqm)	17 (24.3)	53 (75.7)		14 (20)	56 (80)		13 (18.6)	57 (81.4)		12 (17.1)	58 (82.9)		22 (31.4)	48 (68.6)		9 (12.9)	61 (87.1)	
**Type Role played in the Company**																		
Operative	48 (42.1)	66 (57.9)	0.052	40 (35.1)	74 (64.9)	0.073	41 (36)	73 (64)	***0.033***	38 (33.3)	76 (66.7)	***0.024***	55 (48.3)	59 (51.7)	***0.011***	28 (24.6)	86 (75.4)	***0.005***
Technical-Professional	10 (38.5)	16 (61.5)		8 (30.7)	18 (69.3)		8 (30.8)	18 (69.2)		6 (23.1)	20 (76.9)		13 (50)	13 (50)		4 (15.4)	22 (84.6)	
Management	19 (25)	57 (75)		15 (19.7)	61 (80.3)		14 (18.4)	62 (81.6)		12 (15.8)	64 (84.2)		21 (27.6)	55 (72.4)		5 (6.6)	71 (93.4)	
**Territory**																		
Northern macro-area of Province of Bari	46 (35.4)	84 (64.6)	0.921	41 (31.5)	89 (68.5)	0.346	35 (26.9)	95 (73.1)	0.372	36 (27.7)	94 (72.3)	0.466	54 (41.5)	76 (58.5)	0.902	22 (16.1)	108 (82.9)	0.921
Province of Barletta Andria Trani	31 (36.1)	55 (63.9)		22 (25.6)	64 (74.4)		28 (32.6)	58 (67.4)		20 (23.3)	66 (76.7)		35 (40.7)	51 (59.3)		15 (17.4)	71 (82.6)	
**Do you receive specific training on food safety?**																		
Yes. regularly	35 (23)	117 (77)	***<0.001***	27 (17.8)	125 (82.2)	***<0.001***	26 (17.1)	126 (82.9)	***<0.001***	23 (15.1)	129 (84.9)	***<0.001***	41 (27)	111 (73)	***<0.001***	13 (8.6)	139 (91.4)	***<0.001***
Yes. occasionally	35 (62.5)	21 (37.5)		30 (53.6)	26 (46.4)		31 (55.4)	25 (44.6)		28 (50)	28 (50)		42 (75)	14 (25)		19 (33.9)	37 (66.1)	
No	7 (87.5)	1 (12.5)		6 (75)	2 (25)		6 (75)	2 (25)		5 (62.5)	3 (37.5)		6 (75)	2 (25)		5 (62.5)	3 (37.5)	
**Do you think it is adequate?**																		
Yes	25 (20.2)	99 (79.8)	***<0.001***	23 (18.6)	101 (81.4)	***<0.001***	19 (15.3)	105 (84.7)	***<0.001***	18 (14.5)	106 (85.5)	***<0.001***	29 (23.4)	95 (76.6)	***<0.001***	10 (8.1)	114 (91.9)	***<0.001***
Quite adequate	28 (48.3)	30 (51.7)		21 (36.2)	37 (63.8)		24 (41.4)	34 (58.6)		19 (32.8)	39 (67.2)		31 (53.4)	27 (46.6)		11 (19)	47 (81)	
Not entirely adequate	18 (66.7)	9 (33.3)		15 (55.6)	12 (44.4)		15 (55.6)	15 (44.4)		16 (59.3)	11 (40.7)		22 (81.5)	5 (18.5)		12 (44.4)	15 (55.6)	
I don’t know how to answer	4 (100)	0		2 (50)	2 (50)		3 (75)	1 (25)		1 (25)	3 (75)		4 (100)	0		2 (50)	2 (50)	
Not existing	2 (66.7)	1 (33.3)		2 (66.7)	1 (33.3)		2 (66.7)	1 (33.3)		2 (66.7)	1 (33.3)		3 (100)	0		2 (66.7)	1 (33.3)	
**Training method**																		
In person	46 (31.1)	102 (68.9)	***0.007***	38 (25.7)	110 (74.3)	0.056	34 (23)	114 (77)	***0.001***	33 (22.3)	115 (77.3)	0.082	50 (33.8)	98 (66.2)	***0.004***	22 (14.9)	126 (85.1)	0.178
At a distance	9 (47.4)	10 (52.6)		6 (31.6)	13 (68.4)		9 (47.4)	10 (52.6)		5 (26.3)	14 (73.7)		11 (57.9)	8 (42.1)		4 (21.1)	15 (78.9)	
Both modes	12 (33.3)	24 (66.7)		11 (30.6)	25 (69.4)		11 (30.6)	25 (69.4)		11 (30.6)	25 (69.4)		18 (50)	18 (50)		6 (16.7)	30 (83.3)	
I don’t know how to answer	10 (76.9)	3 (23.1)		8 (61.6)	5 (38.4)		9 (69.2)	4 (30.8)		7 (53.9)	6 (46.1)		10 (76.9)	3 (23.1)		5 (38.5)	8 (61.5)	
**How often do you receive traning?**																		
Annual	29 (25)	87 (75)	***0.001***	20 (17.2)	96 (82.8)	***0.001***	24 (20.7)	92 (79.3)	***0.013***	23 (19.8)	93 (80.2)	0.098	32 (27.6)	84 (72.4)	***<0.001***	11 (9.5)	105 (90.5)	***0.001***
Biennial	13 (33.3)	26 (66.7)		16 (41)	23 (59)		12 (30.8)	27 (69.2)		11 (28.2)	28 (71.8)		19 (48.7)	20 (51.3)		6 (15.4)	33 (84.6)	
Triennial	10 (62.5)	6 (37.5)		9 (56.3)	7 (43.7)		9 (56.3)	7 (43.7)		8 (50)	8 (50)		13 (81.2)	3 (18.8)		7 (43.7)	9 (56.3)	
Every four years	22 (56.4)	17 (43.6)		15 (38.5)	24 (61.5)		15 (38.5)	24 (61.5)		12 (30.8)	27 (69.2)		22 (56.4)	17 (43.6)		10 (25.6)	29 (74.4)	
Other	3 (50)	3 (50)		3 (50)	3 (50)		3 (50)	3 (50)		2 (33.3)	4 (66.7)		3 (50)	3 (50)		3 (50)	3 (50)	
**Have you ever participated in specific training courses on allergen risk management (e.g., gluten)?**																		
No	44 (44.9)	54 (55.1)	***0.010***	37 (37.8)	61 (62.2)	***0.011***	39 (39.8)	59 (60.2)	***0.002***	30 (30.6)	68 (69.4)	0.158	50 (51)	48 (49)	***0.008***	23 (23.5)	75 (76.5)	***0.024***
Yes	33 (28)	85 (72)		26 (22)	92 (78)		24 (20.3)	94 (79.7)		26 (22)	92 (78)		39 (33.1)	79 (66.9)		14 (11.9)	104 (88.1)	
**Have you ever heard of the Food Safety Culture?**																		
No	33 (61.1)	21 (38.9)	***<0.001***	27 (50)	27 (50)	***<0.001***	31 (57.4)	23 (42.6)	***<0.001***	25 (46.3)	29 (53.7)	***<0.001***	39 (72.2)	15 (27.8)	***<0.001***	21 (38.9)	33 (61.1)	***<0.001***
Yes	44 (27.2)	118 (72.8)		36 (22.2)	126 (77.8)		32 (19.7)	130 (80.2)		31 (19.1)	131 (80.9)		50 (30.9)	112 (69.1)		16 (9.9)	146 (90.9)	
**On a scale of 1 (a little) to 10 (a lot), how much do you think your work can affect food safety?**																		
Low (1–3)	4 (80)	1 (20)	***0.001***	4 (80)	1 (20)	***<0.001***	5 (100)	0	***<0.001***	4 (80)	1 (20)	***<0.001***	4 (80)	1 (20)	***<0.001***	4 (80)	1 (20)	***<0.001***
Medium (4–7)	34 (48.6)	36 (51.4)		29 (41.4)	41 (58.6)		34 (48.6)	36 (51.4)		26 (37.1)	44 (62.9)		40 (57.1)	30 (42.9)		17 (24.3)	53 (75.7)	
Very much (8–10)	39 (27.7)	102 (72.3)		30 (21.3)	111 (78.7)		24 (17)	117 (83)		26 (18.4)	115 (81.6)		45 (31.9)	96 (68.1)		16 (11.3)	125 (88.7)	

**Table 3:** Logistic regressions analysis.

			*[95% Conf. Interval]*	
***() Number of observations***	***Odds ratio***	***p > |z|***	***Inferior***	***Superior***
***Leadership (212)***				
				
*Knowledge of food safety culture*				
*Ref: No*				
*Yes*	2.78	0.011	1.28	6.04
*Adequacy training in food safety*				
*Ref: Yes*				
*Quite adequate*	0.38	0.011	0.18	0.80
*Not entirely adequate*	0.31	0.045	0.10	0.97
				
*Food safety training periodicity*				
*Ref: Yes, regular*				
*Yes, occasional*	0.43	0.043	0.19	0.97
*No*	0.07	0.029	0.01	0.77
				
*Length of service*				
*Ref: <10 years*				
*>10 years*	2.68	0.006	1.32	5.43
***Communication (216)***				
				
*Knowledge of food safety culture*				
*Ref: No*				
*Yes*	2.95	0.005	1.39	6.28
				
*Food safety training periodicity*				
*Ref: Yes, regular*				
*Yes, occasional*	0.31	0.001	0.15	0.63
*No*	0.09	0.008	0.01	0.53
				
*Length of service*				
*Ref: <10 years*				
*>10 years*	3.82	<0.001	1.80	8.07
***Commitment (216)***				
				
*Food safety training periodicity*				
*Ref: Yes, regular*				
*Yes, occasional*	0.20	<0.001	0.09	0.41
*No*	0.06	0.002	0.01	0.37
				
*What role do you play?*				
*Ref: Operational*				
*Management*	2.71	0.013	1.23	5.97
				
*Adequacy training in food safety*				
*Ref: Yes*				
*Quite adequate*	0.37	0.011	0.18	0.79
				
*Knowledge of food safety culture*				
*Ref: No*				
*Yes*	3.94	<0.001	1.88	8.26
***Risk Awareness (216)***				
				
*Food safety training periodicity*				
*Ref: Yes, regular*				
*Yes, occasional*	0.31	0.004	0.13	0.69
*No*	0.08	0.001	0.02	0.36
				
*Adequacy of training in food safety*				
*Ref: Yes*				
*Quite adequate*	0.37	0.013	0.17	0.81
*Not entirely adequate*	0.25	0.011	0.08	0.73
				
*What role do you play?*				
*Ref: Operational*				
*Management*	2.56	0.021	1.16	5.62
***Resources (209)***				
				
*Food safety training periodicity*				
*Ref: Yes, regular*				
*Yes, occasional*	0.15	<0.001	0.07	0.33
				
*Adequacy of training in food safety*				
*Ref: Yes*				
*Quite adequate*	0.44	0.032	0.21	0.93
				
*Knowledge of food safety culture*				
*Ref: No*				
*Yes*	4.49	<0.001	2.03	9.93
				
*What role do you play?*				
*Ref: Operational*				
*Management*	2.94	0.005	1.39	6.25
				
*How often do you receive training?*				
*Ref: Yearly*				
*Triennial*	0.18	0.021	0.04	0.77
***General food safety climate (216)***				
				
*Length of service*				
*Ref: <10 years*				
*>10 years*	3.33	0.015	1.26	8.79
				
*How often do you receive training?*				
*Ref: Yearly*				
*Triennial*	0.22	0.019	0.06	0.78
*Other (One-off, five-year, etc.)*	0.15	0.047	0.02	0.98
				
*What role do you play?*				
*Ref: Operational*				
*Management*	4.39	0.015	1.33	14.51
				
*Knowledge of food safety culture*				
*Ref: No*				
*Yes*	4.44	0.001	1.79	11.00
				
*Have you received food safety training?*				
*Ref: Yes, regular*				
*No*	0.06	0.003	0.01	0.40
				
*Adequacy of training in food safety*				
*Ref: Yes, adequate*				
*Not entirely adequate*	0.28	0.019	0.10	0.81

## Data Availability

The original contributions presented in the study are included in the article. Further inquiries can be directed to the corresponding author.

## Abbreviations

The following abbreviations are used in this manuscript:

FSC	Food Safety Culture
FSMS	Food Safety Management System
FBO	Food Business Operator
HACCP	Hazard Analysis and Critical Control Points
HCW	Healthcare Worker
